# Recent Progress in Extracellular Vesicle-Based Carriers for Targeted Drug Delivery in Cancer Therapy

**DOI:** 10.3390/pharmaceutics15071902

**Published:** 2023-07-07

**Authors:** Yaqin Tang, Xingyou Liu, Meng Sun, Su Xiong, Nianting Xiao, Jianchao Li, Xiao He, Jing Xie

**Affiliations:** 1 Chongqing Key Laboratory of Medicinal Chemistry and Molecular Pharmacology, Chongqing University of Technology, Chongqing 400054, China; 2 College of Pharmaceutical Sciences, Southwest University, Chongqing 400715, China

**Keywords:** extracellular vesicles, cancer therapy, drug delivery, nanomedicine

## Abstract

Extracellular vesicles (EVs) are small, membrane-based vesicles released by cells that play a critical role in various physiological and pathological processes. They act as vehicles for transporting a variety of endogenous cargo molecules, enabling intercellular communication. Due to their natural properties, EVs have emerged as a promising “cell-free therapy” strategy for treating various diseases, including cancer. They serve as excellent carriers for different therapeutics, including nucleic acids, proteins, small molecules, and other nanomaterials. Modifying or engineering EVs can improve the efficacy, targeting, specificity, and biocompatibility of EV-based therapeutics for cancer therapy. In this review, we comprehensively outline the biogenesis, isolation, and methodologies of EVs, as well as their biological functions. We then focus on specific applications of EVs as drug carriers in cancer therapy by citing prominent recent studies. Additionally, we discuss the opportunities and challenges for using EVs as pharmaceutical drug delivery vehicles. Ultimately, we aim to provide theoretical and technical support for the development of EV-based carriers for cancer treatment.

## 1. Introduction

Extracellular vesicles (EVs) are released from cells through paracrine or autocrine secretion under physiological and pathophysiological conditions and can be isolated from various body fluids [1]. Structurally, EVs consist of a phospholipid bilayer and various cyst contents, which are membranous vesicles actively released by cells. Furthermore, EVs also play an important role in many physiological and pathological processes, enabling intercellular communication by acting as a vehicle for a variety of endogenous cargo molecules such as RNA, proteins, carbohydrates, and lipids [2,3,4,5,6,7,8]. Generally, depending on their formulation, size, or function, EVs can be classified into exosomes from the endocytic pathway, microvesicles from the plasma membrane, and apoptotic bodies resulting from vesicles and apoptosis [9]. In recent years, an increasing number of studies have revealed that EVs function as natural mediators of intercellular communication, and their potential for organ or cell targeting, excellent biocompatibility, site specificity, and enhanced intranuclear body escape have increased interest in their diagnostic and therapeutic applications for various diseases including cancer [10,11,12,13].

In the past few decades, despite substantial contributions to cancer therapy, cancer is still without question one of humanity’s biggest killers, and there is no effective treatment to surmount this challenge [14]. Thus, substantial amounts of research are aimed at innovating anticancer drugs, such as small-molecule drugs, nucleic acids, proteins, and other nanomaterials [15,16]. However, there are still many gaps hindering cancer treatment efficiency including low bioavailability, high toxicity, low specificity, instability, enzymatic degradation, rapid clearance, and tumor cell resistance [17,18,19,20]. To overcome these drawbacks, researchers have devised synthetic nanocarriers to facilitate controlled and targeted drug release, mitigate systemic cytotoxicity, and minimize adverse effects [21,22,23,24]. Such carriers have gained widespread use in cancer therapy. One of the most commonly utilized carriers is liposomes, versatile drug transporters capable of concurrently encapsulating various drugs and integrating them with finely tuned stimulus-responsive strategies, ultimately achieving synergistic cancer treatment. Analogously, the multifunctioning of EVs in transferring bioactive molecules between cells suggests these particles can be used as therapeutic agents or drug delivery vehicles, including both water-soluble and water-insoluble varieties. Furthermore, owing to their organotypic properties and tumor-targeting capabilities, EVs offer a desirable alternative to synthetic nanoparticles, which makes them beneficial for the accumulation of therapeutic drugs at the affected area after being administered systemically. In addition, EVs have the potentiality to flee from clearance by the host immune system and subsequently to pass through physiologic handicaps due to specific membrane-based protein expression and small size [25,26,27]. Together, these unique biological characteristics make EVs one of the most promising carriers for targeted drug delivery in cancer therapy [28,29,30,31,32].

This article aims at summarizing the latest research findings on EVs in recent years and reviews the research on Evs as therapeutic carriers for cancer treatment. First of all, the biological characteristics of Evs (mainly about exosomes, microvesicles, and apoptotic bodies) are well summarized, especially their biogenesis and isolation techniques for Evs. We also discussed the working principles, advantages, and drawbacks of these separation methods. In addition, the general drug loading and functionalization of Evs are summarized pre- and post-separation individually. Furthermore, the application of EV-based carriers and their effects on cancer therapy in recent years are reviewed. It also addresses the progress of Evs in the efficacy of small-molecule drugs, nucleic acids, proteins, and other nanomaterials. Finally, we summarize the recent advances of Evs and put forward our prospective idea about Evs as nanovehicles for delivering therapeutic agents with an emphasis on their applications in cancer therapy, which will help us better understand the current research progress and future directions of EV-based carriers for targeted drug delivery in cancer treatment.

## 2. Classification of Evs

### 2.1. Biogenesis of Exosomes

In 1981, Trams et al. discovered a group of vesicle-like structures with diameters 40–1000 nm smaller than those of polyvesicles, identified by transmission electron microscopy (TEM) [33]. Later, Johnstone et al. discovered vesicle-like structures during the maturation of reticulocytes and isolated them from sheep reticulocytes by centrifugation [34]. These vesicle-like structures were then named exosomes for the first time. Essentially, exosomes are vesicles of an endosomal origin, which pinch off the surface of the plasma membrane via outward budding with a size range of ~40 to 160 nm (average ~100 nm) in diameter. Sequential invagination of the plasma membrane ultimately results in the formation of multivesicular bodies, which can intersect with other intracellular vesicles and organelles, contributing to diversity in the constituents of exosomes [35]. Exosome contents not only mirror the composition of the donor cell but also reflect a regulated sorting mechanism [36]. The contents of exosomes are mostly proteins, lipids, and RNA, which can stably exist in blood, urine, cerebrospinal fluid, saliva, ascites, vaginal secretions, and other body fluids [37]. In addition, exosomes, as the mediators of signal transmission between cells, can provide autocrine, paracrine, and endocrine types of signals. In 1996, it was reported that exosomes secreted by dendritic cells and B lymphocytes may have functions related to immune regulation and can be used as carriers of antitumor immune response compounds and participate in the whole process of tumor development [38].

### 2.2. Biogenesis of Microvesicles

Microvesicles are 100–1000 nm vesicles released from the plasma membrane to outgoing buds and to the extracellular environment by fission [39]. They are widely distributed in a variety of body fluids, including urine, peripheral blood, and peritoneal effusion. The function and composition of MVs are related to the cells of origin, including tumor cells, stem cells, immune cells, and endothelial progenitor cells. The microvesicles are shed directly from the extramembrane buds. However, the shedding process is associated with lipid and protein composition and the rearrangement of plasma membrane molecules at the level of calcium ions [40]. There are some signal pathways involved in this process, such as the calpain-dependent pathway. The calpain-dependent pathway stimulates calcium intracellular flow by an agonist and activates thiol protease and calpain in the cytoplasm to move to the cell membrane. Then, they bind with phosphate ester on the membrane and generate calmodulin by calcium-regulated conformational change. Activated calmodulin cleaves α-actin and talin filaments, allowing cytoskeleton proteins to be separated, thus causing MV release [41]. So, intracellular Ca^2+^ increasing may cause the asymmetric phospholipid distribution in the plasma membrane to change, leading to actin cytoskeleton maintenance depolymerization and promoting microvesicle shedding.

### 2.3. Biogenesis of Apoptosis Bodies

Apoptosis is a kind of programmed death, which occurs in billions of cells every day. Apoptotic bodies are specifically produced in the process of apoptosis and can wrap the cytokines secreted in the process of apoptosis [42]. Functionally, apoptotic bodies can be recognized, phagocytized, and cleared by macrophages, fibroblasts, and specific phagocytes (xerophytes), which also can mediate the transfer of biomolecules including microRNAs and proteins between cells to aid intercellular communication [43]. However, compared with exosomes and microvesicles, apoptotic bodies are relatively large vesicles, with a diameter of 800–5000 nm [44], and the composition of apoptotic bodies is in direct contrast to exosomes and microvesicles. Unlike exosomes and microvesicles, apoptotic bodies contain intact organelles, chromatin, and some glycosylated proteins. Thus, one observes proteins associated with the nucleus (i.e., histones), mitochondria (i.e., HSP60), Golgi apparatus, and endoplasmic reticulum (i.e., GRP78). Moreover, the proteomic characteristics of apoptotic bodies and cell lysates are similar, while the proteomic characteristics of exosomes and cell lysates are significantly different [45]. In addition, due to the large and uneven volume of apoptotic bodies, most studies mainly focus on exosomes and microvesicles, though a few studies are using apoptotic bodies as therapeutic nanomedicine [46].

## 3. EV Isolation Methods

EVs, as circulating phospholipid vesicles secreted by cells, can play an important role in intercellular communication and the establishment of tumor microenvironments. However, EVs exist in complex biological fluids and contain a variety of pollutants, so it is necessary to use some appropriate separation and enrichment methods to obtain relatively pure EVs [47]. In this review, seven kinds of separation and enrichment techniques commonly used in recent years are discussed: “standard” ultracentrifugation, density-gradient centrifugation, co-precipitation, size-exclusion chromatography, ultrafiltration, immunoaffinity enrichment, and field flow fractionation. The working principles, advantages, and drawbacks of these techniques are described in Table 1. Newer fluid systems are also being developed to enhance efficiency and make the process faster [48].

### 3.1. Ultracentrifugation

Ultracentrifugation (UC) is recognized as the current gold standard for the enrichment and purification of exosomes [57]. It can be used for large-scale preparation of EVs from different biological fluids, and EVs can also be isolated from a large number of samples with less reagent consumption. In general, low-speed centrifugation (e.g., 300–2000 g) was used to remove cells and apoptotic fragments. High-speed centrifugation (e.g., >10,000 g) is suitable for separating EVs from cellular metabolites or protein aggregates. However, although centrifugation is simple to operate, the purity of EVs obtained is relatively low. In addition, there are other disadvantages; for instance, the required instruments are very expensive, the separation time is long, and the centrifugal force being too large may affect the integrity of the separation results [49]. Therefore, many other methods are also being studied in full swing.

### 3.2. Density-Gradient Ultracentrifugation

Density-gradient ultracentrifugation (DGUC) is a technique to improve particle separation efficiency according to the buoyant density of particles. In recent years, two methods have been used to form gradients: a continuous density gradient (centrifugation or pre-formation) and a stepwise gradient (density increases in a discrete manner), namely, a sucrose cushion. A higher purity of EV preparation is obtained by the standard protocol of density-gradient ultracentrifugation compared to classical ultracentrifugation. In addition, the method has been shown to be superior to classical ultracentrifugation and commercial kits in terms of EV purity, EV protein, and RNA quantity [54]. Currently, density-gradient ultracentrifugation is commonly used for the isolation of microvesicles. However, this approach not only results in a significant loss of EVs, but also is complex, laborious, time-consuming (up to 2 days), and requires expensive equipment [50].

### 3.3. Co-Precipitation

Co-precipitation (CP) has been a relatively emerging technique in recent years which is mainly based on the formation of a mesh polymer net that captures EVs in the 60–180 nm size range and then shapes them into particles at low centrifugation speeds. As of now, a variety of commercial precipitation kits can be used to precipitate EVs from different biological fluids. These kits are based on the super-hydrophilic volumetric exclusion polymer polyethylene glycol (PEG), which separates EVs based on their reduced solubility in PEG solutions [58]. Polymer precipitation results in a higher yield of extracellular vesicles than ultrafiltration, and this method requires no specialized equipment and is simple to implement. However, PEG isolation is not pure; it also precipitates many other materials like protein aggregates and larger vesicles, which makes this method unsuitable for many downstream analyses [59]. Precipitation also preserves the structural integrity and biological function of EVs. However, it is difficult to separate polymers such as PEG from EVs, which may affect the results of subsequent studies, and these kits are expensive for large-scale use [52]. Therefore, as an independent EV isolation method, the utility of CP is limited.

### 3.4. Size-Exclusion Chromatography

Size-exclusion chromatography (SEC) separation is based on particles of different sizes passing through different elution profiles of porous polymers, forming stationary phases (also known as gel filtration substrates or resins), and passing through mobile phases of SEC columns [60]. When the sample enters the gel, small molecules diffuse into the pores, while large molecules are eluted directly. Consequently, larger molecules exit the column earlier than small molecules, which makes it possible for a molecule’s residence time to correlate with its size. This separation method has been recently applied to vesicle isolation to obtain purified EVs from a complex biological culture medium [48]. It can be applied to a variety of biological fluids, including cell culture media, plasma, serum, urine, milk, saliva, nasal lavage fluid, synovial fluid, cerebrospinal fluid, ascites, and tears [61]. Several studies have demonstrated the superiority of SEC over conventional EV separation techniques, with cell culture- and plasma-derived EVs produced by SEC having better functioning compared to those produced by UC [62,63,64]. The recovery rate of plasma-derived EVs separated by differential UC is also negatively affected by the high viscosity of the plasma. SEC also yielded higher-quality cell culture-derived EVs with less protein contamination and fewer composition and structural changes than EVs isolated using precipitators such as PEG and protein organic solvent precipitation [65]. Although SEC can effectively remove relatively small proteins from plasma, its main disadvantage is that it is difficult to co-separate with other lipoproteins because they have the same size range as EVs and are not easily separated from EVs efficiently in the column. In addition, we found that SEC isolates relatively smaller EVs than UC, resulting in different proteomes. SEC’s self-packaging is also cumbersome, which reduces the reliability of the method [53].

### 3.5. Ultrafiltration

Ultrafiltration (UF) is a method of separating the components of a sample based on their sizes; this technique relies on the use of membranes with specified pore diameters to isolate particles of a pre-determined size range. Larger particles are eliminated first by using filters with pore diameters of 0.8 and 0.45 µm, leaving a relatively exosome-rich filtrate. Smaller vesicles are then eliminated from the filtrate by using membranes with pores smaller than the desired exosomes (0.22 and 0.1 µm), causing them to pass into a waste eluate [66]. UF is mainly used to separate proteins and other unwanted contaminants from EV samples. The method is not limited by sample size, and the process is simple, which means it can be used as a supplement for separating large particles and microvesicles and exosomes by ultracentrifugation [67]. However, due to the interaction between the vesicles and the membrane, the membrane acts as a binding surface for EVs and proteins in the solution, creating aggregates and effectively blocking the pores. This reduces the efficiency of the UF method and reduces the purity and yield of the isolated EVs [68,69].

### 3.6. Immunoaffinity Enrichment

In general, EV isolation by immunoaffinity enrichment (IE) mainly utilizes the immune affinity interactions between proteins (antigens) in EVs and their antibodies, as well as specific interactions between receptors and ligands. Owing to its simplicity and immunoaffinity capture, immunoaffinity enrichment is an attractive approach at present. Ideally, EV biomarkers for immunoisolation are membrane-bound, lack soluble counterparts, and are only expressed or highly concentrated on the surface of extracellular vesicles from specific biological sources [70]. However, the requirement of large sample size, the limited sensitivity, and the time-consuming protocol reduce the method’s clinical practicability [55].

### 3.7. Field Flow Fractionation

Field-flow fractionation (FFF) is an emerging size-based EV separation technology, in which asymmetric flow fractionation (AsFlFFF or AF4) is the most commonly used FFF sub-technology. In FFF, the separation system is not a column but a parallelepiped channel in which the force field is perpendicular to the sample stream to achieve separation based on different sizes and molecular weights [71]. In AsFlFFF, small EVs move away from the bottom of the channel due to their higher diffusion coefficient, while large EVs move closer to the stacking wall [72]. Coupled with a fraction collector, EV subpopulations can also be collected for additional studies. One of the advantages of AsFlFFF for EV separation is gentle fractionation because there are no shear forces from the stationary phase deteriorating the particles, as in the case of SEC. In addition, AsFlFFF allows buffer exchange with the EV formulation buffer, which is important especially in potential therapeutic applications of fractionated EV subpopulations. However, the EVs isolated by AsFlFFF have been diluted and may need to be pre-enriched for further study. In addition, in order to avoid self-correlation and overload effects, only small amounts of samples can be injected, so the method is not suitable for handling large numbers of samples [56].

## 4. Drug Loading of EVs

EVs, a novel natural drug carrier, possess a lipid bilayer which can protect a drug from degradation in the blood and circumvents safety issues arising from the use of cationic synthetic nanoparticle carriers in vivo. EVs can also cross the blood–brain barrier easily and are being tested regarding whether drug-loaded vesicles could be used to target glioblastoma [73]. However, efficient encapsulation and targeted delivery of therapeutic drugs, such as small-molecule drugs, and nucleic acids (siRNA, mRNA, and miRNA), which are currently the main therapeutic agents, are the main challenges. In particular, compared to chemical drugs, nucleic acid drugs currently face more obstacles, such as intracellular susceptibility to enzymatic degradation, effective targeted delivery, immune stimulation, and off-target effects. Therefore, EVs may be a promising drug delivery system for exogenous small molecules and gene therapy. From this perspective, we will provide an overview of the techniques for loading EVs with drugs, both prior to and after isolation.

### 4.1. Pre-Loading

Pre-loading primarily involves loading cargo (nucleic acids, proteins, and small-molecule compounds) into cells or carrier materials before or during the generation of EVs. For example, cocultivation of HEK293 and COS-7 cells with vectors that can express miR-16, -21, -143, -146a, or -155 is performed so that relevant miRNAs can be overexpressed in the cells and combined with an endogenous RNA secretion mechanism to trigger the active release of EVs. In receptor COS-7 cells, EVs encapsulating miRNAs still have the ability to induce gene silencing [41,42]. Encapsulation of relevant therapeutic factors or small molecules prior to EV isolation enables the EV membrane to maintain its integrity. Nevertheless, the amount of drugs loaded into EVs is challenging due to the different transfection efficiencies of various RNAs and the viabilities of different cell lines.

Abundant multipurpose EVs can be secreted from Plasmodium falciparum when grown in its natural host red blood cells (RBCs) [74]. Vorselen et al. characterized the structure of RBC-EVs using atomic force microscopy and found that their material properties were extremely similar to those of liquid liposomes [75]. In addition, EVs were purified and modified from erythrocytes and then conjugated with peptides, nanobodies, and monoclonal antibodies to form peptide/antibody-fitted RBC-EVs, which finally demonstrated good biocompatibility and non-immunogenicity [76].

Mesenchymal stromal cells (MSCs) are powerful regenerative cells that exert therapeutic effects through paracrine mechanisms and produce numerous secretions, including EVs. Human-bone-marrow MSC-secreted EVs induce the proliferation of tubular epithelial cells after acute kidney injury through mRNA transfer, while enhancing their anti-apoptotic effects [77]. Additionally, generous MSC-secreted EVs were observed in a rat model of acute myocardial infarction, which improved functional recovery by promoting angiogenesis to protect cardiac tissue from ischemic injury [78]. Borgovan et al. found that MSC-secreted EVs could inhibit the proliferation of de novo acute myeloid leukemia (AML) cell lines in vitro through an apoptotic mechanism, and further investigation manifested the therapeutic potential of MSC-EVs as a monotherapy or adjuvant therapy [79]. Furthermore, exogenous small molecules cultured in MSCs can induce the secretory production of EVs. Chu et al. studied the neuroprotective and anti-inflammatory effects of EVs derived from pretreatment of MSCs with the gas transmitter hydrogen sulfide (H_2_S), and the results indicated that H_2_S-EVs could induce miR-7b-5p expression and improved cognitive impairment in neonatal mice [80].

When tumor cells undergo apoptosis, cell membranes will be contracted and divided; therefor, functional biomolecules are actively packaged into vesicles to efficiently produce membrane-encapsulated apoptotic vesicles (ABs) [81]. Dongyang Zhao et al., used the chemotherapeutic drug camptothecin (CPT) to induce apoptosis; the remaining drug was encapsulated into apoptotic vesicles released from dead or dying cells, while CSSP NPs were prepared by self-assembly of the heterodimeric prodrug CPT-ss-PR104A to exhibit good tumor penetration effects, superior tumor growth inhibition, and anti-metastatic ability in vitro and in vivo under AB-mediated proximity effects [82].

### 4.2. Post-Loading

In general, the process of post-loading is divided into two steps: exosome separation by the above techniques and drug fusion. So, the simplest approach to loading therapeutic drugs is to mix EVs with free drugs thoroughly or supplement them with mild sonication, especially for hydrophobic compounds. Paclitaxel (PTX) is a highly hydrophobic drug commonly used for chemotherapy. For instance, by simply incubation at room temperature, PTX can be encapsulated into EVs. Additionally, another method is to sonicate the mixture followed by further incubation at 37 °C for 1 h; the loading efficiency of EVs with PTX was increased by nearly 10-fold after sonication [83]. Cationic complexes can also mediate interactions with negatively charged EVs via cationic charges, and electrostatic interactions allow the complexes to be potentially immobilized on the vesicle surface or taken up by EVs via endocytosis or fusion with the membrane.

The natural barrier of EVs, the lipid bilayer, limits the passive loading of therapeutic drugs into EVs. Thus, electroporation came into being, which stimulates spontaneous pore formation in membranes mainly by compensating for voltage changes triggered by electrical signals, and it is an effective method to achieve nucleic acid drug loading after EV isolation [84]. In addition, for hydrophilic compounds, electroporation may be superior to passive mixing [85]. Xu et al. utilized a CRISPR/Cas9 system for plasmid targeting of the human cMyc gene, delivered either systemically or locally, by BioRad Gene Pulser (Bio Rad Inc., Hercules, CA, USA), enabling selective accumulation in tumor tissue and ultimately allowing for in vivo gene editing [86]. Osteikoetxea et al. developed a novel method for loading CRISPR/Cas9 into extracellular vesicles (EVs) using a combination of cryptochrome 2, CD9 antibodies, and specifically modified lipids, resulting in efficient loading and highly functional delivery of Cas9 molecules [87]. However, related studies found that electroporation may result in aggregation or fusion of EVs, forming insoluble siRNA aggregates [88], and the efficiency of electroporation may be significantly lower than anticipated. Therefore, there is an urgent need to discover alternative methods for loading large molecules, such as siRNAs, into EVs to maximize their therapeutic potential.

Therefore, the simple incubation-induced fusion of EVs/liposomes or other synthetic objects not only has the properties of synthetic carriers but also the natural properties of EVs, leading to high loading and loading rates, specific targeting, and better endosomal escape. However, the negative charge carried by most liposomes may limit effective fusion between EVs and liposomes due to electrostatic interactions. To address this issue, Piffoux et al., developed a fusion of EVs with polyethylene glycol (PEG)-functionalized liposomes, creating intelligent biosynthetic hybrid vectors with adaptable activity and drug delivery properties [89].

In addition to the above methods, high loading efficiency of EVs can also be achieved by other means such as treatment with saponin, extrusion, or freeze–thaw cycles, as demonstrated by Haney et al., in an in vivo/vitro model of Parkinson’s disease by the protein-encoding plasmid DNA transfection of macrophages. Their study utilized several different ways to load peroxidase into EVs, and EVs were found to maintain high loading efficiency after sonication and extrusion or saponin-mediated treatment, effectively preventing peroxidase degradation [90].

## 5. Functionalization of EVs

### 5.1. Pre-Functionalization

#### 5.1.1. Gene Engineering

Genetic engineering techniques can be utilized to introduce coding and non-coding oligonucleotides into cells and integrate them into extracellular vesicles (EVs) to promote gene expression or regulate transcription in recipient cells. Born et al. employed mesenchymal stem cells transfected to overexpress long non-coding RNA HOX transcript antisense RNA (HOTAIR-MSCs) for the secretion and generation of EVs [91]. The abilities of angiogenesis and wound healing were also demonstrated in a diabetic mouse model. Transgenic proteins can also be integrated into EVs as fluorescent markers or targets; for example, when utilizing highly expressed interleukin-12 (IL-12) or TGF-β1 shRNA for genetic engineering of MC38 colon cancer cells, the modified MC38 cells, upon modification, secrete exosomes and microvesicles [92]. In addition, cardiac-targeted EVs with better targeting ability (CTP-EVs) were prepared by cotransfection of HEK293 cells with CTP-FLAG-LAMP2b-HA and mCherry-CD81 plasmids. In HEK293T cells, EVs are generated by transfecting parental cells with a fusion plasmid containing the albumin-binding peptide ABP-Lamp2b [93]. However, the peptide–Lamp2b fusion protein is susceptible to degradation by protease in endosomes, and thus close monitoring of the target, drug, or marker is required to ensure the integrity of the fraction when employing genetic engineering strategies.

#### 5.1.2. Metabolic Engineering

Metabolic engineering allows the incorporation of metabolite analogs into cellular biosynthesis by introducing functional groups such as azides into EVs for bio-orthogonal reactions. Tu et al., used the metabolic precursor tetra-acetylated N-azidoacetyl-D-mannosamine (Ac4ManNAz) self-assembled with azide groups to form nanoparticles (Az-NPs) that were taken up by tumor cells and expressed azide groups on the plasma membrane, followed by secretion of azide-containing EVs and their transfer to deep tumor regions. Subsequently, by conjugating dibenzocyclooctyne-modified chlorin e6 (DBCO-Ce6), they were combined with photodynamic therapy to improve treatment efficiency [94]. Lim et al., also used Ac4ManNAz metabolic glycoengineering (MGE) to modify exogenous azide moieties on the cell surface and subsequently used dibenzocyclooctyl polyglycolylated hyaluronic acid (DBCO-PHA) with a specific binding affinity for CD44-overexpressed tissues to enable effective PHA-EVs to accumulate effectively into the target tissue or tumor [95].

#### 5.1.3. Source Cell Alteration Engineering

The last strategy for modifying parental cell membranes is liposome-based cell engineering, where a liposome or micelle that can fuse with the cytoplasmic membrane is selected, and EVs are introduced by exchanging membrane components. He et al., constructed an EV-mediated self-propelled liposome through membrane fusion liposomes, which maintained the structural integrity and stability of EVs and transferred fused lipids with functionalization directly to the plasma membrane of the recipient cells, achieving therapeutic or targeting effects on cancer cells in a more effective and controlled manner [96]. Reginald-Opara et al., investigated the effect of pH on liposome transcytosis and found that non-pH-sensitive liposomes could use the EVs secretion pathway to cross the blood–brain barrier for their brain endothelial cell transcytosis, whereas pH-sensitive liposomes facilitated cytoplasmic delivery [97].

### 5.2. Post-Isolation

#### 5.2.1. Physical Modification

The physical modification of extracellular vesicles (EVs) following their isolation is commonly achieved through techniques such as fusion with liposomes, insertion of lipophilic fractions into membranes, and molecular adsorption onto their surfaces. For instance, researchers such as Zhou et al. have successfully utilized a new EV called a membrane-hybridized lipid nanovesicle (LEV) for efficient delivery of siRNA via Golgi and endoplasmic reticulum pathways, resulting in up to a sevenfold increase in siRNA transfection efficiency [98]. Meanwhile, Zachary et al. have fused HER2 overexpressing BT-474 cells to EVs via plasma membrane fusion, allowing for a specific loading of HER2 antibodies onto the surface of MDA-MB-231 cells [32]. Anti-HER2 antibody-coupled paclitaxel liposomes were then utilized for the targeted delivery of HER2. Similarly, Piffoux et al., have randomized a functionalized liposome triggered by polyethylene glycol (PEG) to create a functionalized vector delivery platform [89].

#### 5.2.2. Chemical Modification

Covalent coupling with relevant specific targeting peptides remains the most common approach for the chemical modification of EVs. For instance, researchers have used covalently coupled epidermal growth factor receptor (EGFR)-targeting peptides or anti-EGFR nanobodies, causing them to accumulate in EGFR-positive cancer cells for targeted therapy [13]. Moreover, a pH concentration gradient can be created inside and outside the cell by EVs’ membrane protonation to enhance efficient and stable loading of nucleic acid drugs such as miRNA, siRNA, and single-stranded DNA (ssDNA) [99]. Ye et al., have exploited the ability of ApoA-I peptide to molecularly recognize phospholipids on the lipid bilayer of EVs to modify EVs with methotrexate-loaded LDL-targeting peptide, significantly improving cellular uptake and penetration into the deeper regions of a tumor [100]. Another common modification technique involves the introduction of azide–alkyne cycloaddition reactions using bio-orthogonal reactions. For example, Li et al., have used azide groups to modify EVs generated from tumor cells for efficient in vivo tumor imaging. Rare-earth-doped EVs emitting strong near-infrared II (NIR-II) nanoparticles were labeled with DBCO-EVs that undergo copper-free click chemistry in vivo, and it was found that trans-bio-orthogonal mediated EVs also have great potential in tumor imaging [101].

## 6. EVs as Drug Delivery Nanovectors

**Figure 1:**
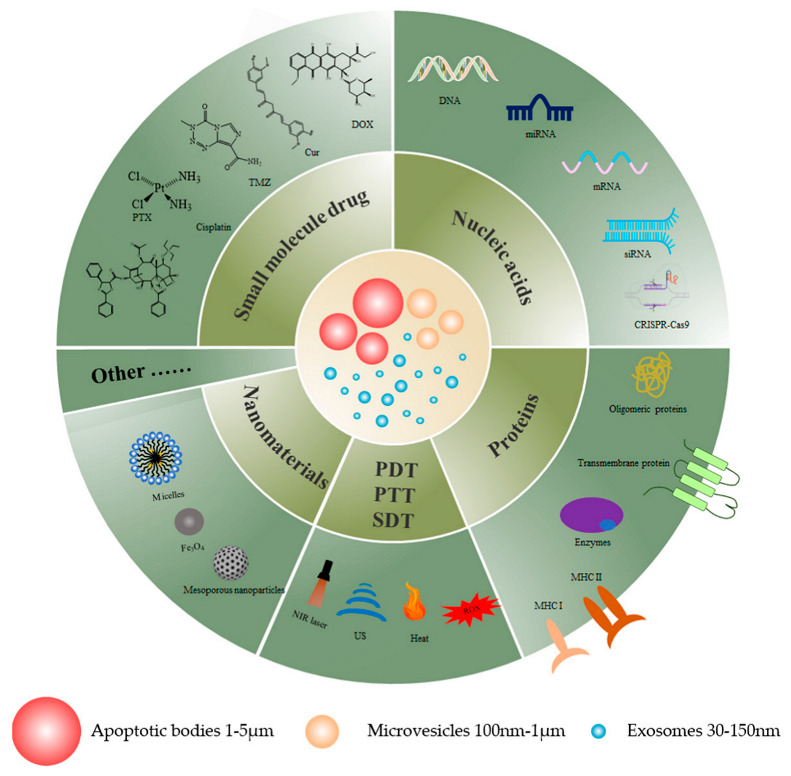
A graphical illustration of EVs as drug delivery nanovectors for cancer therapy.

EVs are small vesicles with membrane structures that include exosomes, microvesicles, and apoptotic bodies of EVs [102] which are considered to be an ideal vehicle for drug delivery due to their non-immunogenicity, low toxicity, and biocompatibility. This review details the drug delivery effects of extracellular vesicles as drug delivery vehicles for cancer therapy. Figure 1 displays a graphical representation of EVs as drug delivery nanovectors, and Table 2 summarizes the use of EVs as nanovectors for therapeutic agents.

### 6.1. Small-Molecule Drugs

Small-molecule drugs as the main tools in cancer treatment have achieved certain results in clinical practice. However, these drugs are prone to problems such as drug resistance and serious side effects in the treatment process. Thus, aiming to solve these problems, researchers have begun to explore novel drug delivery vehicles, and extracellular vesicles are one of the natural delivery systems with great potential, which can not only improve the bioavailability of drugs, but also enhance the therapeutic efficacy and reduce the pain of patients.

#### 6.1.1. Chemotherapy Drugs

Chemotherapeutic drugs are widely recognized as the most commonly used small-molecule drugs, particularly when it comes to first-line treatment of oncology patients. Exosomes are a highly researched subcategory of EVs which are vesicles enclosed in membranes and secreted by nearly all cell types. Delivery of chemotherapy drugs via exosomes has been shown to reduce systemic toxicity and to be more therapeutic than monotherapy [133]. The targeting of exosomes was also verified. Exosomes from tumor cells encapsulate Doxil and can be returned to the corresponding tumor tissue after systemic administration. These findings highlight the great potential of exosomes as carriers for the delivery of therapeutic agents in cancer therapy and demonstrate the advantages of tumor cell exosomes for targeting tumor sites [134]. Bioinspired mimetic exosome nanovesicles, products of the extrusion of tumor cells after incubation with paclitaxel, also showed a strong antitumor effect following in vivo delivery [135]. In addition, when EVs deliver chemotherapeutic agents in synergy with other anticancer agents, the anticancer effect is more significant than when they are administered alone. For example, EVs from human lung cancer cells, when administered in combination with oncolytic virus and paclitaxel (PTX), may improve tumor resistance in naked rats [30]. The EVs derived from the macrophages M1 (M1 EV), charged with bis [2,4,5-trichloro-6-(pentoxicarbonyl)phenyl] oxalate (CPPO), the chloroprotein e6 (Ce6), and the former drug aldubicine (DOX-EMCH), are also capable of inducing several anticancer effects together [129].

Other than tumor cells, EVs from other sources have also been reported to possess antitumor properties. EVs derived from MSCs have significant potential as a guided anti-tumor drug delivery platform because of their strong tendency to migrate toward tumor sites, showcasing their remarkable bioengineering potential. Pascucci et al. were the first researchers in Italy to suggest that the use of exosomes from mesenchymal stem cells as carriers of paclitaxel (PTX) could significantly enhance the antitumor effects of PTX in vivo [136]. In studies involving the administration of PTX, the antitumor efficacy of chemotherapeutic agents can be enhanced by modifying the host cells that produce exosomes. For instance, Wang et al. utilized the pro-inflammatory properties of activated M1 macrophages to create a pro-inflammatory environment via the release of pro-inflammatory factors, which enhances the antitumor effect of PTX delivered by M1-exo and increases caspase-3 expression in breast cancer cells [137]. The EVs of embryonic stem cells are well adapted as vectors due to their wireless self-renewal capability. The exosomes of embryonic stem cells have also been proven to have antitumor properties [138] but without the homing effect of EVs of tumor origin. Therefore, to improve their targeting capability, it is possible to engineer exosomes to have a specific target by expressing various proteins on their surface. Zhu et al. have demonstrated that embryonic stem cell exosomes modified by cancer chemotherapy targeting ligand peptide (RGDyk) improve the ability of exosomes to cross the BBB, thereby significantly improving the in vivo antitumor efficacy of PTX [139]. In addition, Liu et al. have developed an EV delivery system derived from HEK293T cells (hEVs) that target tumor sites. The hEVs are lipid-linked with HA, which enables the specific targeting of CD44 and inhibition of P-GP expression to reduce drug efflux. In an MDR (multidrug resistance) tumor model, lipHA-hEV-mediated delivery of DOX exhibited high tumor specificity, which increased DOX accumulation at the tumor site, and inhibited tumor growth without significant chemical toxicity in organs [140]. Tian et al. developed a targeted antitumor drug delivery system using immature dendritic cell-derived exosomes fused with iRGD peptides and targeting αV integrins. The purified iRGD exosomes were then loaded with DOX using electroporation. The study demonstrated that the iRGD exosomes efficiently delivered DOX to αv integrin-positive cancer cells, exhibiting potent antitumor effects [141].

In the field of cancer drug resistance, EV-mediated delivery of chemotherapeutic drugs has shown promising results. Studies have shown that exosomes, released from macrophages and loaded with PTX using sonication, can overcome P-GP-mediated drug resistance in MDCK cells with greater cytotoxicity than free drugs [142]. Building on this work, researchers have developed an engineered exosome delivery system modified with aminoethyl anisamide-polyethylene glycol (AA-PEG) to target lung cancer cells. The results showed higher drug-loading capacity and better therapeutic efficacy. TMZ, a second-generation oral alkylating agent, can also reduce tumor resistance through exosome encapsulation [106].

As a natural carrier, EVs have the potential to traverse the BBB, making them an ideal vehicle for the treatment of brain tumors. However, the current ability of EVs to cross the BBB remains limited. Bai et al. developed a delivery system that enhances the ability of EVs to cross the BBB and enhances brain targeting through the use of focused ultrasound (FUS). This delivery system was shown to successfully load DOX and significantly inhibit tumor growth, with no side effects observed in animal models [112].

**Figure 2:**
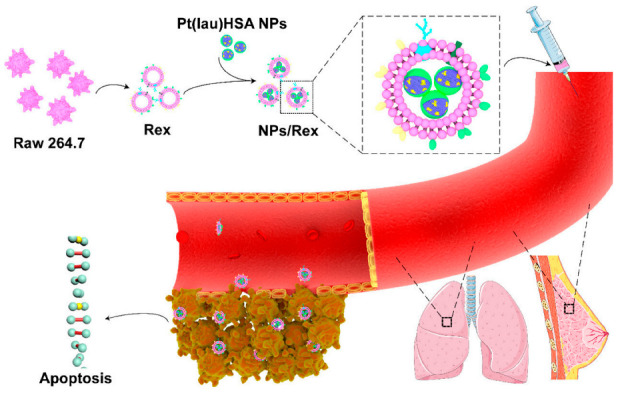
Schematic illustration of the Pt(lau)HSA NP-loaded exosome platform (NPs/Rex) for efficient chemotherapy of breast cancer [143]. Copyright 2019, American Chemical Society.

Xiong et al. (Figure 2) used macrophage-derived exosomes to encapsulate functionalized platinum (Pt), which largely reduced the systemic toxicity of chemotherapeutic drugs. This study synthesized nanoparticles (NPs) using nanoprecipitation of lauric acid-functionalized Pt(IV) prodrug (Pt(lau)), human serum albumin (HSA), and phospholipids. The NPs were then encapsulated by macrophage-secreted exosomes, termed RAW exosome (Rex), to form the NPs/Rex delivery system. At the cellular level, Rex enhanced the cellular uptake of Pt and resulted in high toxicity against multiple cancer cells, particularly breast cancer cells. Furthermore, in animal models, Rex exhibited strong tumor-homing capabilities, accumulated in breast orthotopic tumors and lung metastatic nodules, and demonstrated potent antitumor efficacy [143].

In recent years, although exosomes have shown promising results as carriers of small-molecule drugs in cancer therapy, other types of vesicles such as apoptotic bodies and microvesicles have also been found to exhibit similar effects. Cell membrane-derived microparticles (MPs) are a subgroup of EVs that can be used as natural delivery systems to treat cancer. MPs (TMPs) derived from tumor cells are used to transport methotrexate (TMPs-MTX), a chemotherapy drug that has been shown to significantly inhibit the growth of MEP [144].

#### 6.1.2. Immune Small-Molecule Drugs

The immune small molecules in EVs, as immunomodulatory carriers, provide fundamental theoretical support for mutual information transfer between cells and communication between cells and their environment and bring about important applications for life science research.

**Figure 3:**
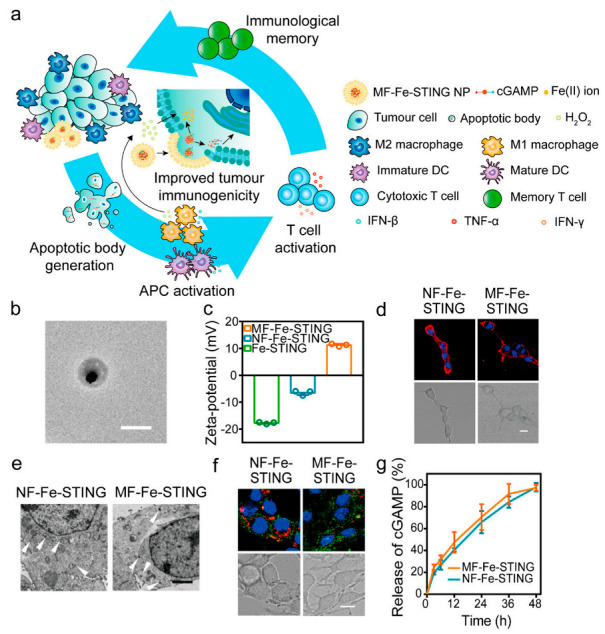
Characterization of MF−Fe−STING NPs. (**a**) Schematic illustration of the intracellular delivery strategy. (**b**) TEM images of MF−Fe−STING NPs (scale bar: 200 nm). (**c**) Zeta potentials of Fe−STING NPs, NF−Fe−STING NPs, and MF−Fe−STING NPs. (**d**) CLSM images of 4T1 murine breast tumor cells after 20 min of incubation with DiI-loaded NF−Fe−STING NPs and MF−Fe−STING NPs, respectively (scale bar: 10 μm). DiI (red) and the nucleus (blue) in confocal images. (**e**) TEM images of 4T1 cells receiving 30 min of incubation with NF−Fe−STING NPs and MF−Fe−STING NPs, demonstrating evidence of the different uptake pathways between membrane and nonfusogenic formulations (white arrowhead) (Scale bar: 2 μm). (**f**) CLSM images of 4T1 cells incubated with 8−Dy547−cGMP-loaded NF−Fe−STING NPs and MF−Fe−STING NPs for 2 h and subsequent treatment with Lysotracker Red stain (scale bar: 10 μm). Nucleus (blue), lysosome (red), and 8−Dy547−cGMP (green) in confocal images. (**g**) Time−dependent cGAMP release curves of NF−Fe−STING NPs and MF-Fe-STING NPs [113]. Copyright 2022, American Chemical Society.

The homing ability of EVs and their ability to carry endogenous substances from host cells make them excellent natural carriers. Huang et al. developed a specific EV loaded with Hiltonol (a TLR3 agonist) and an immunogenic cell death (ICD) inducer, human neutrophil elastase (ELANE), as a DC vaccine. The EVs secreted by Hela cells have specific targeting for tumor cells and can deliver drugs precisely to the tumor site. This concept can be extended to any tumor cell by loading ICD inducers and immune adjuvants onto EVs for cancer therapy [145]. EVs can also help other immune molecules to escape from the immune system; for instance, PD-L1 expression on EVs derived from glioblastoma cells has been shown to inhibit T cell activation through binding to PD-1 on the T cell surface [146]. The small-molecule-targeted drug lapatinib is a tyrosine kinase receptor inhibitor when loaded on exosomes which exhibits superior antitumor effects to free drugs [110]. Interferon gene stimulator (STING) agonists have emerged as a promising immunotherapy that can trigger effective innate immunity, but their delivery capacity is poor. To overcome this matter, Peng et al. (Figure 3) developed a delivery platform based on the cooperative promotion of apoptotic bodies (ABs) derived from tumor cells that are easily engulfed by antigen-presenting cells (APCs) and the Fenton reaction and STING-activating nanoparticles. By loading ABs with Fe(II) ions, STING-activating nanoparticles, and cGAMP, an exogenous adjuvant, they enhanced the ability of ABs to be engulfed by APCs, leading to a multifaceted antitumor therapy [113]. In addition, cytidylyl phosphate guanosine (CpG)-modified gold–silver nanorods (AuNR) have been incubated with tumor donor cells to produce apoptotic bodies (AB) loaded with the nanomedicine (AuNR-CpG/AB), which actively targets tumors through the natural homing effect of ABs. By combining tumor accumulation promoted by ABs, immune stimulation promoted by CpG, and tumor antigen release induced by hyperthermia, effective immunotherapy can be achieved, effectively preventing tumor metastasis and recurrence [147].

### 6.2. Nucleic Acids

For tumor treatment, nucleic acid drugs have more advantages than traditional drugs due to their unique mechanism of action, such as strong specificity, abundant gene targets, not easily producing drug resistance, lasting efficacy, and so on [148]. However, nucleic acid drugs have disadvantages in vivo, such as poor bioavailability, easy degradation, and off-target effects. Therefore, such drugs need effective delivery platforms to deliver them to the target site for disease treatment [149]. EVs open up new pathways for gene therapy with their unique advantages.

#### 6.2.1. miRNA

MicroRNAs (miRNAs) are a type of single-stranded non-coding RNA with small molecular weights that play important and complex roles in tumor proliferation and development, including cell proliferation, apoptosis, tumor invasion, and epithelial–mesenchymal transition [150]. An miRNA can bind to the 3′ untranslated region of its target gene and then regulate the target gene by controlling its translation process or stability.

Among the many studies on miRNA delivery, the most widely used method is exosomes derived from mesenchymal stem cells (MSC), which offer great advantages in terms of biocompatibility, safety, and targeting. Using exosome delivery of miR-9-3p, miR-139-5p, and miR-138-5p from MSCs to treat bladder cancer, MSCs-Exo can successfully deliver miRNAs to bladder cancer tissue and block the proliferation, migration, and invasive abilities of cancer cells, effectively inhibiting tumor progression [114,151,152]. Studies have shown that miRNA-21 levels are significantly elevated in glioblastoma [153]. Kim et al., modified the membranes of exosomes with T7 peptide (transferrin receptor-binding peptide) and Lamp-2b and then used electroporation to load AMO-21 into the exosomes. T7-Exo effectively delivered it to the brain and reduced miR-21 levels, thereby inhibiting tumor growth [118]. Similarly, Lee et al., prepared exosome-mimetic cell membrane nanovesicles (CMNVs) by extrusion, modified them with T7 peptide, and then delivered AMO21c to inhibit miR-21 expression in GBM, also showing better targeting while slowing down tumor growth [154].

In addition to the above studies, a variety of other miRNAs are delivered by exosomes (including miR-204-5p, miR-146b, miR-497, and miR-159). By affecting the expression of related genes, exosomal miRNAs can effectively inhibit tumor growth and metastasis, as well as modulate the sensitivity of tumor cells to drugs [115,117,131,155].

#### 6.2.2. siRNA

siRNAs can knock down the expression of target genes in a sequence-specific manner by mediating the degradation of targeted mRNAs. Compared to small-molecule therapeutics and monoclonal antibody drugs, siRNAs have an advantage because they perform their functions by strictly following Watson–Crick base pairing with mRNAs [156].

Cancer progression is related to the upregulation of anti-apoptotic proteins such as PLK1 (Polo-like kinase 1), KRAS, and cell growth factor. Exosomes were originally thought to be natural carriers of siRNA in vitro. siRNA exosome therapies targeting tumor cells have been aimed at downregulating the expression of these oncogenes. KRAS mutations are among the most common gene mutations in tumors, causing uncontrolled cell proliferation and thus a high risk of cancer, and are associated with pancreatic, lung, and colon cancers. Previous studies have shown the efficacy of siG12D-LODER in patients with locally advanced pancreatic cancer [157]. Therefore, a recent study transfected siG12D-LODER into exosomes, which were then injected into mice as a model of pancreatic cancer. The results showed that the siRNA-containing EVs were able to successfully target downregulation of the KRAS gene in cancer cells, which considerably extended the lifespans of mice, while also effectively suppressing tumor growth and promoting apoptosis in tumor tissues. This study also compared the method with liposomal delivery of siRNA, and the results of animal experiments showed that the targeting and antitumor effects of liposomes were inferior compared to those of EVs with siRNA, further demonstrating the advantages of EVs [158]. In another study, an Exo-An2-siRNA targeting delivery system was constructed for the treatment of GBM by modifying the membranes of exosomes and loading them with siRNA. This safe and effective functionalized exosome provides a new therapeutic strategy for GBM therapy [121]. Tumor-derived extracellular vesicles (TDEVs) have a strong intercellular material transfer function in the tumor microenvironment. The constructed siCDK1@LEVs inherit the intercellular messaging ability of TDEV membrane proteins and the efficient encapsulation of siRNA by liposomes, targeting the “homing” properties of parental cells and intracellular “highway” transport via the Golgi–endoplasmic reticulum pathway. The “homing” properties of the targeting parental cells and the intracellular “highway” transport through the Golgi–endoplasmic reticulum pathway greatly increase drug aggregation at the tumor site [98].

#### 6.2.3. mRNA

With the successful development of mRNA vaccines for COVID-19, there is now an active trend to develop mRNA drugs for cancer treatment. mRNA could be used to give patients drugs that match the characteristics of their individual cancer cells, promising to help patients who have not responded to conventional treatments.

Scorch death induction in tumors has promising applications in cancer immunotherapy. GSDMD-N mRNA was encapsulated within EVs, and the EV membrane was engineered and modified for delivery into HER2+ breast cancer cells to directly induce scorch death to enhance antitumor immunity [159].

Yang et al., used cellular nanoperforation (CNP) to infiltrate high levels of mRNA into exosomes, and CNP produced up to 50 times more mRNA transcripts; electroporation increased mRNA transcripts by more than 103-fold. In GBM model mice, tumor growth was significantly inhibited, and the survival rate of mice was improved [122]. Wang et al., engineered EVs to construct EXO-DEPTs, which were then used to deliver functional exogenous mRNAs that specifically targeted HER2+ cells and were shown to block tumor growth in vivo [160]. Therapeutic mRNA vaccines are of great research value regarding tumor immunity. EVs from the recombinant plasmid-transformed BL21 (DE3) *Escherichia coli*, which has an innate immunostimulatory function, have also been used as an mRNA delivery platform for personalized tumor vaccines. The nanocarrier platform has a “Plug-and-Display” function that allows the design of personalized cancer vaccines based on their tumor antigens. Subcutaneous injection of EVs carrying ovalbumin or ADPGK mRNA in mice significantly arrested the progression of melanoma [161].

#### 6.2.4. CRISPR-Cas9

Gene editing is a new therapeutic method developed in recent years which is expected to treat cancer by regulating gene expression and correcting gene mutations. Among these techniques, CRISPR/Cas9 is a promising treatment for cancer as a genetic tool that can be used to treat a wide range of diseases through DNA splicing with ease of use and precision [162]. Kim et al., employed exosomes loaded with CRISPR/Cas9 to suppress the expression of poly (ADP-ribose) polymerase-1 (PARP-1) and trigger apoptosis in ovarian cancer cells [124]. By using EVs as a vector for CRISPR/Cas9 delivery, McAndrews et al., demonstrated in a mouse model a delivery platform for CRISPR/Cas9 DNA that inhibits oncogenic KrasG12D in vitro and tumor growth in vivo [163]. Exosomal gene editing nanoparticles (exosome RNP) obtained by loading Cas9 RNP into liver fibroblast-derived exosomes can successfully deliver RNP into target cells to produce efficient gene-editing effects [164].

#### 6.2.5. ASO

In nucleic acid drug therapy, antisense oligonucleotides (ASO) are also a hot topic of research. mRNA can be targeted by ASOs in a highly specific manner to downregulate the expression of disease-causing proteins. ASO drugs can also increase the amount of protein in diseases caused by the under-expression of proteins [165]. In contrast to siRNA, ASO not only acts on mRNA, but also regulates some noncoding RNAs, most of which only work outside the nucleus. An engineered exosome has been developed to deliver an antisense oligonucleotide (ASO) targeting STAT6 (exoASO-STAT6), which selectively silences the expression of STAT6 in tumor-associated macrophages (TAMs). In preclinical models of colorectal cancer and hepatocellular carcinoma, treatment with exoASO-STAT6 resulted in a significant inhibition of tumor growth [126]. In short, ASOs are able to restore endogenous proteins to normal levels.

### 6.3. Protein

Proteins with biological functions and enzymes with therapeutic effects suffer from poor delivery due to cell membrane limitations. In contrast, EVs are an ideal protein delivery vehicle because they can stably release their loading substances through multiple pathways. EVs can be used to carry a wide range of proteins, including enzymes, cytokines, antigens, hormones, antibodies, receptors, and more. The diversity and specificity of the proteins delivered by exosomes allow for targeted delivery to different types of cells and therefore a wide range of applications.

In 2011, extracellular vesicles utilized rabies virus glycoprotein as a targeting peptide for systemic cancer delivery therapy, and in later studies, researchers identified additional targeting peptides. Other targeting peptides, such as the αγ integrin-specific peptide iRGD, can be attached to exosomes as well [166]. Fusion of a fragment of the target cancer receptor interleukin 3 (IL3) with Lamp2b allows for exosomal targeted cancer therapy [167]. Koh et al., developed exosomes loaded with signal-regulatory protein α (SIRPα) using a CD47 blockade strategy. The SIRPα exosomes were obtained by transfecting HEK293T cells with the SIRPα gene and then centrifuging them. SIRPα binds to CD47, sending a “don’t eat me” signal and leading to immune recognition. The engineered exosomes carrying SIRPα variants were able to control macrophage phagocytosis in vitro and effectively inhibit tumor growth in vivo [168]. Researchers further developed a ferritin nanocage, FN-SIRPα, and found that exosomes as a delivery platform had more advantages than other carriers [169]. Other studies have reported the use of exosomes as carriers, loading natural PH20 hyaluronidase into exosomes and helping natural PH20 hyaluronidase to enter the tumor microenvironment more easily and deplete overexpressed hyaluronan (HA) for tumor therapy [170]. Tumor necrosis factor (TNF)-related apoptosis-inducing ligand (TRAIL) is another anticancer protein that, when expressed on the surface of EVs, has been shown to overcome drug resistance in some cancer cells. Antibody drugs can also be delivered by EVs, as demonstrated in experiments where the encoding genes of antihuman CD3 UCHT1 scFv antibody and antihuman HER2 trastuzumab scFv were transfected into Expi293F cells [171], resulting in SMART-Exos that effectively and selectively induced tumor-specific immunity against HER2-expressing tumors. These findings demonstrate the potential of EVs as a powerful tool for immune modulation and cancer therapy [172]. Exosomes loaded with PDL1-blocking single-chain variable fragments (scFv) have also been shown to have an immunotherapeutic effect on tumors [173].

In addition to antibody drugs, the research team has developed a nanovaccine called Exo-OVA using antigens found in tumors. The vaccine incorporates new antigens from ADPGK in MC-38 tumors and M16 and M10 in B27F30 melanoma tumors into EVs. In corresponding tumor models, the Exo-OVA vaccine has demonstrated antitumor immune effects [174].

### 6.4. Others

#### 6.4.1. Photothermal Therapy

In recent years, photothermal therapy (PTT) and photodynamic therapy (PDT) have developed rapidly in the treatment of cancer. This type of treatment works by introducing photosensitizers (PSs) that convert absorbed light energy into heat or produce toxic reactive oxygen species (ROS), thereby causing damage to cancer cells. Jang et al. [175] loaded a photosensitizer (Ce6) in a self-assembled manner into membranes derived from tumor recombinant exosomes to obtain Ce6-R-Exo and then used photoacoustic imaging (PA) to show that laser irradiation of Ce6 activated PDT, while some immune cytokines were gradually released, effectively inhibiting tumor growth and metastasis. Studies have shown that PTT and PDT have great potential for use in combination with chemotherapy or immunotherapy in the field of oncology treatment and are more effective than either method alone [176]. Xia et al., treated gastric cancer (GC) with fluorescent dye-induced PTT in combination with chemotherapy [177]. First, they isolated EVs from HEK-293 cells with nanobodies against CDH17 on their surface. Then, the team engineered EVs with the near-infrared fluorescent dye ICG and chemotherapeutic agents. In a GC model, CDH17-EVs were able to rapidly image and show significant PTT effects after irradiation, in addition to inducing immunogenic cell death as well as inducing macrophages to polarize from M2 to M1. In addition, one study combined genetically engineered exosomes with drug-carrying thermosensitive liposomes to design a hybrid nanovesicle, and in vitro and in vivo experiments showed that this drug delivery strategy was able to combine with PTT and completely eliminate tumors while inducing a strong immune response.

**Figure 4:**
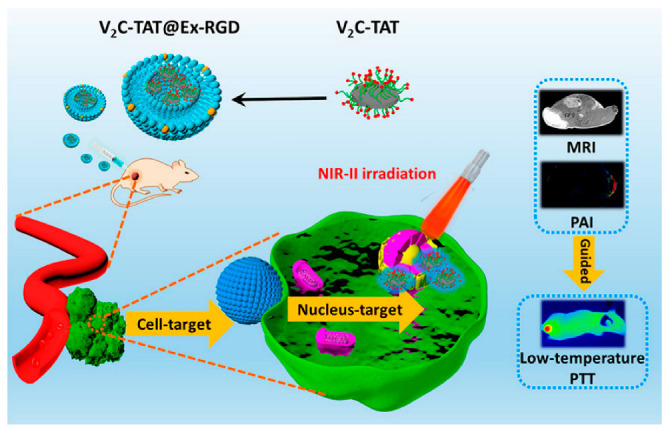
Engineered Exosome-Mediated Near-Infrared-II Region V_2_C Quantum Dot Delivery for Nucleus-Targeting Low-Temperature Photothermal Therapy [178]. Copyright 2019, American Chemical Society.

A recent study has shown that when vanadium carbide quantum dots (V_2_C QDs) and PTA are integrated with an engineered exosome vector, the system is capable of low-temperature nucleus-targeted PTT in the NIR-II region to achieve effective tumor killing (Figure 4). This strategy offers several advantages over PTT, including minimal adverse reactions, high resistance to hyperthermia, and excellent depth of penetration [178]. In conclusion, photothermal therapy has broad application prospects in the field of anticancer drug delivery.

#### 6.4.2. Sonodynamic Therapy

Sonodynamic therapy (SDT) is a new type of oncology treatment that has been developed in recent years. Ultrasound has a powerful penetrating ability. The principle of STD is to use this ability to penetrate deep into the tissues and then activate a sonosensitizer to produce ROS to kill tumor cells. An exosome-based strategy has been used for local delivery of sonosensitizers (Ce6) and resiquimod (R848) to the tumor site. It was shown that ExoCe6+R848 activated DCs and reversed the tumor-suppressive microenvironment in xenograft models after ultrasound irradiation [179]. Guided ultrasound (US1) was employed to facilitate the targeted accumulation of EXO-DVMS in the tumor area, followed by the application of therapeutic ultrasound (US2) to carry out SDT. The results of the study showed that EXO-DVDMS achieves controlled drug release by ultrasound, and ROS production is enhanced. In conclusion, it promotes simultaneous imaging and tumor metastasis inhibition in vivo [180].

#### 6.4.3. Combination Therapy

Combination therapy offers significant advantages over monotherapy, including improved efficacy, dose reduction, regulation of drug resistance, and reduction of toxic side effects. At the same time, the combined effects of multiple drugs can be greater than the sum of the efficacies of the individual drugs, and this “synergistic effect” can increase the effectiveness of cancer treatment to a greater extent [181,182].

**Figure 5:**
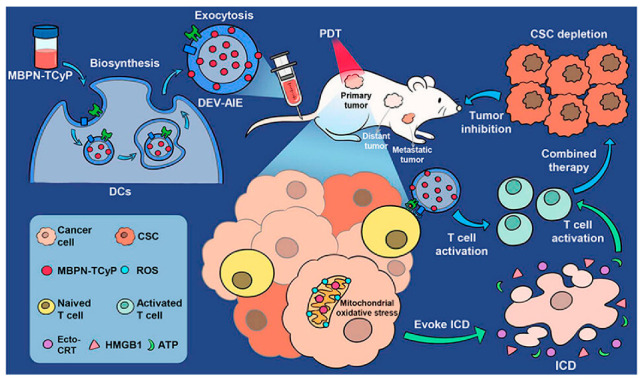
Biosynthetic Dendritic Cell-Exocytosed Aggregation-Induced Emission Nanoparticles for Synergistic Photodynamic Immunotherapy [185]. Copyright 2022, American Chemical Society.

Wang et al., constructed a CCA-M1EVs drug delivery system for the treatment of GBM by encapsulating AQ4N after membrane modification of M1 macrophage-derived vesicles (M1EVs) with CPPO and Ce6. This delivery platform achieved a powerful therapeutic effect with the synergistic effects of immunomodulation, CDT, and hypoxia-activated chemotherapy [183]. Another study also used M1EVs to encapsulate CPPO, Ce6, and Dox-EMCH in a synergistic, three-mode anticancer treatment with engineered self-activating photo-EVs, and this synergy produced effective anticancer treatment and reduced drug side effects [129]. Conventional SDT may be poor in terms of therapeutic efficacy due to the BBB and hypoxic TME. Nanoparticles formed by the adsorption of catalase (CAT)-loaded silica nanoparticles (CAT@SiO_2_) and a sonosensitizer (ICG) are encapsulated by macrophage exosomes which are functionally modified by AS1411 nucleic acid aptamers. This method has high BBB penetration and is effective in overcoming tumor hypoxia. In vivo experiments demonstrated that these engineered exosomes promoted the efficacy of SDT [184]. By utilizing DCs as cellular responders to biosynthesize DEV-AIE NPs, the synergistic effect between DEV immunotherapy and MBPN-TCyP PDT resulted in not only the elimination of the primary tumor but also the stimulation of a systemic tumor-specific cytotoxic T cell response, leading to complete suppression of untreated distant and metastatic tumors (Figure 5). In addition, immunocompetent DEV-AIE showed significant inhibition of CSCs in 4T1 and CT26 solid tumors [185]. CSSP NPs were prepared by combining camptothecin (CPT), PR104A, and disulfide bond self-assembly. CPT induced apoptosis, resulting in ABs containing CPT and PR104A. The AB-mediated neighboring effect promotes tumor infiltration by CSSP NPs, and thereby all tumor cell subgroups are eliminated [82]. A recent study combined chemical/genetic/photothermal therapies with Fe_3_O_4_@PDA and then added engineered exosomes to produce complex delivery systems for accurate cancer diagnosis and treatment [186]. In the future, precision and combination therapies are likely to be very important directions to explore in the field of oncology treatment.

## 7. Conclusions and Prospects

Due to the advantages of EVs, such as biocompatibility and strong bioactivity, recently, researchers have made great efforts to conduct studies of exosome extraction verification and applications in drug delivery. EVs originating from natural resources can be regarded as universal and efficient vectors compared with traditional artificial nanoparticles. Due to the properties of EVs, including a closed-loop structure and special protein expression on the surface, EVs could prevent cargos from premature release and degradation in the extracellular environment. Moreover, EVs have the targeting delivery ability inherited from the original parental cells. EV-assisted delivery could help cargo to overcome biological barriers such as tumor blood vessels for efficient aggregation in tumor tissues. Compared to synthesized nanoparticles, EVs have good properties such as low biological toxicity and great biocompatibility.

Despite great achievements having been made in studies about EVs, there still exist several difficulties that limit the further application of EV-mediated drug delivery for clinical therapy. The main difficulty is how to achieve scaled-up, uniform, and stable EVs. Currently, bioreactors and streamlined purification protocols via microfluidic devices are the important strategies for the efficient and improved production of EVs. Moreover, it is worth noting that quality control is crucial in the large-scale production of EVs for application in clinical therapy. In addition, the second difficulty is how to increase the loading efficiency of cargo of EVs, which affects the application prospects of EVs in biotherapy. A novel method should be developed for acquiring great efficiency in drug encapsulation of EVs without using large amounts of them. EVs have several kinds of proteins and functional immune molecules on their membrane which will trigger an immune response, inducing the rapid clearance of EV-based delivery. Therefore, EVs need to be modified and engineered to avoid potential system toxicity and side effects, simultaneously evaluated by measuring their pharmacokinetics and pharmacodynamics.

EVs have been used as drug delivery vectors for preliminarily exploring their potential application in clinical therapy and trials. EVs are employed to transport therapeutic proteins, nucleic acids, and small-molecule drugs, which are widely discussed and have indicated great potential value in cancer treatment. Furthermore, novel approaches are applied to design and modify EVs to enhance their targeting, biotherapeutic, and cargo-loading efficiency. Moreover, researchers have the ability to improve the production of EVs with stable, uniform, and large-scale properties by advanced methodologies. The process of obtaining clinical-grade EVs has been in progress, but further efforts are needed. In addition, among EVs, ABs are promising; although there are currently relatively few application findings, they also play an important role in various diseases, which expands the great potential value of ABs in biomedical applications such as immunotherapy, disease detection, and drug delivery. Studies have indicated that ABs have the ability to decrease pathological conditions, and they show great advantages in drug delivery. Altogether, these delivery vectors inherited from natural mechanisms have revealed the long circulation, good stability, and biocompatibility of EVs for drug delivery.

## Figures and Tables

**Table 1:** Comparison of EV Enrichment, Separation, and Purification Methods.

Isolation Methods	Working Principle	Advantage	Drawback	Ref.
Ultracentrifugation	Based on the size of EVs. Large ones precipitate earlier, while small ones require greater centrifugal force to precipitate easily.	The most commonly used method; Less reagent consumption; EVs can be isolated from a large number of samples.	High equipment cost; Time-consuming; High- speed centrifugation may damage EVs.	[49]
Density-gradient ultracentrifugation	Based on EV density.	High purity; Good to maintain the activity of EVs.	Complexity; Time-consuming.	[50,51]
Co-Precipitation	Polymer-based precipitators bind to hydrophobic proteins and lipid molecules for co-precipitation to separate EVs.	Easy and simple to handle; Low time requirement.	Low purity and recovery; More heteroprotein; Produces polymers that are difficult to remove.	[52]
Size-Exclusion Chromatography	Based on the size of EV molecules, a porous gel matrix causes separation.	High purity.	Needs special equipment; Time-consuming and laborious.	[53]
Ultrafiltration	Relative division using different interceptions; a sub-mass ultrafiltration membrane is used for selective separation of samples.	Simple and efficient; No sample size limitation; Does not affect EVs’ biological activity.	Low yields; Protein contamination; Deformation of vesicles.	[54]
Immunoaffinity Enrichment	EV surface-specific marker, coated with corresponding antibody; EVs can be isolated by incubating the magnetic beads with EVs.	Simple operation; Does not affect EVs’ morphological integrity; High specificity.	Low efficiency; Not suitable for large quantities; Antibodies are expensive.	[55]
Field-flow fractionation	Macromolecules flow through flat channels, applying force fields perpendicular to the sample flow to achieve separation based on different sizes and molecular weights.	Broad separation range; Wide variety of eluents.	Lengthy duration; Requires fractionation equipment.	[56]

**Table 2:** EVs as nanovectors for therapeutic agents.

Cargo Types		Specific Substances	Extracellular Vesicles Source	Type of Extracellular Vesicles	Loading Method	Effect	Cancer Types	Ref.
Small-Molecule Drugs	Chemotherapy drugs	Doxorubicin	MSC	Exosome	Electroporation	Inhibits tumor growth	Colorectal cancer	[103]
		Paclitaxel	PC-3	Exosome Microvesicle	Co-incubation	Enhances the cytotoxicity of paclitaxel in autologous prostate cancer cells	Prostate cancer	[23]
		Cisplatin	Macrophage cell	Exosome	Co-incubation	Reverses cisplatin resistance, Inhibits tumor growth	Ovarian cancer	[104]
		Curcumin	PANC-1	Exosome	Co-incubation	Induces apoptosis in cancer cells.	Pancreatic cancer	[105]
		Temozolomide	Glioma cells	Exosome	Co-incubation	Reverses TMZ resistance, Inhibits tumor growth	GBM	[106]
		Camptothecin	4T1	Apoptotic bodies	Co-incubation	Enhances tumor growth suppression and antimetastatic ability	Breast cancer	[82]
	Immune small-molecule drugs	TGFβRI kinase inhibitor and TLR7/8 agonist	FBS	Exosome	Electroporation	Inhibits tumor growth	Melanoma and Prostate cancer	[107]
		MHC, CD86, αCD3 Ab, and αEGFR Ab	DC	Exosome	Co-incubation	Activates T cells and increases their killing ability, Inhibits tumor growth	B16-OVA melanoma	[108]
		Anti-CD3/CD28 single-chain variable fragments (scFvs)	HEK293T	Exosome	Transfection	Activates T cells and increase their killing ability	Gastric cancer	[109]
		Lapatinib	MCF10 A	Exosome	Electroporation	Activates T cells and increases their killing ability	Breast cancer	[110]
		A33Ab	LIM1215	Exosome	Co-incubation	Improves tumor-targeting capabilities	Colorectal cancer	[111]
		CpG	EL4	Apoptotic body	Co-incubation	Prevents tumor metastasis and recurrence	lymphoma	[112]
		cGAMP	Breast cancer cell	Apoptotic body	Active loading	Enhances STING activation and an improves tumor-specific antigen presentation ability	Breast cancer	[113]
Nucleic Acids	miRNA	miR-138-5p	ADSCs	Exosome	Lentivirus infection	Inhibits tumor growth	Bladder cancer	[114]
		miRNA-497	HEK293T	Exosome	Chemical transfection	Regulates the growth, migration, and angiogenesis of tumors	Lung cancer	[115]
		miR-199a	AMSC	Exosome	Lentivirus infection	Improves the sensitivity of tumor cells to DOX	HCC	[116]
		miR-146b	MSC	Exosome	Electroporation	Inhibits tumor growth	GBM	[117]
		miRNA-21	HEK293T	Exosome	Electroporation	Inhibits tumor growth	GBM	[118]
	siRNA	siS100A4	Auto logous breast cancer cells	Exosome	Co-incubation and Extrusion	Inhibits tumor growth	Breast cancer	[119]
		siRNA	HEK293T	Exosome	Chemical transfection	Significant tumor growth regression	NSCLC	[120]
		siSTAT3	RAW	Exosome	Ultrasonication and Incubation	Inhibits tumor growth	GBM	[121]
		siCDK1	Sk-hep1	EVs	Electroporation	Inhibits tumor growth	HCC	[98]
	mRNA	PTEN	MEFs and DCs	Exosome	Cellular-nanoporation	Inhibits tumor growth	Glioma	[122]
		5-FC and yCD::UPRT mRNA	HEK-293T	Microvesicle	Co-incubation	Inhibits tumor growth	Glioma	[123]
	CRISPR-Cas9	CRISPR-Cas9	HEK293/SKOV3	Exosome	Electroporation	Induces apoptosis in ovarian cancer. Enhances chemosensitivity to cisplatin.	Ovarian cancer	[124]
		CRISPR-Cas9	HEK293T	EVs	Sonication	Inhibits tumor growth	Liver cancer	[125]
	ASO	ASO-STAT6	HEK293/M2 macrophages	Exosome	Mixing	Inhibits tumor growth	Colorectal cancer and HCC	[126]
Proteins		Transferrin receptor-binding peptide	MDA-MB-231	Exosome	Mixing	Inhibits tumor growth	Breast cancer	[118]
		Tlyp-1	M1macrophage	Exosome	Co-incubation; Electroporation	Inhibits tumor growth	Breast cancer	[127]
		αCD3/αEGFR	M1macrophage	EVs	Electroporation	Inhibits tumor growth	Breast cancer	[128]
		Transferrin receptor-binding peptide	HEK293T	Exosome	Transfection	Improves tumor-targeting capabilities	GBM	[118]
Combination Therapy		CPPO/Ce6/Dox-EMCH	THLG-293T/LG-293T	EVs	Electroporation	Reverses drug resistance in colon cancer.	Colon cancer	[129]
		miR-21/5-FU	HEK293T	Exosome	Co-incubation	Inhibits tumor growth	Breast cancer	[130]
		Dox/Cho-miR-159	THP-15	Exosome	Co-incubation	Powerful ferroptosis promotion in GBM. Inhibits tumor growth	GBM	[131]
		siGPX4/Fe_3_O_4_@mSiO_2_	HEK293T	Exosome	Co-incubation	Inhibits tumor growth	Triple-negative Breast cancer	[132]
		CPT-SS-PR104A	Tumor cell	Apoptotic bodies	Active loading	Inhibits tumor growth	Breast cancer	[82]

## Data Availability

Not applicable.
